# Editorial: Plasticity in Multiple Sclerosis: From Molecular to System Level, from Adaptation to Maladaptation

**DOI:** 10.3389/fneur.2015.00265

**Published:** 2015-12-22

**Authors:** Daniel Zeller, Maria A. Rocca

**Affiliations:** ^1^Department of Neurology, University of Würzburg, Würzburg, Germany; ^2^Neuroimaging Research Unit, Division of Neuroscience, Institute of Experimental Neurology, San Raffaele Scientific Institute, Milan, Italy

**Keywords:** plasticity, adaptation, multiple sclerosis, motor, visual, cognitive reserve, rehabilitation, TMS

**The Editorial on the Research Topic**

**Plasticity in Multiple Sclerosis: From Molecular to System Level, from Adaptation to Maladaptation**

Multiple sclerosis (MS) is an inflammatory disease that affects the central nervous system (CNS) by demyelination and direct axonal injury (1). Decades of research have focused on pathophysiological issues of the disease and, at least for the relapsing-remitting type of MS, have achieved considerable progress with respect to the prevention of relapses and accumulation of MS-related clinical impairment (2). However, only recently there is increasing awareness that the individual course of MS might not only be governed by neuroimmunological properties of the disease but also determined by the innate capacity of the CNS to overcome functional constraints related to MS pathology, i.e., by the patient’s individual resilience. Accordingly, variation in brain plasticity is believed to play a crucial role in explaining interindividual differences with respect to the clinical course as well as to discrepancies between functional impairment and magnetic resonance imaging (MRI) findings in patients with MS (3, 4).

Plasticity occurs at multiple levels in MS, from cells to synapses, from myelin to axons, from individual regions to large-scale brain networks [reviewed in Ref. (5)]. A growing body of evidence supports the notion that the course of MS and its extremely heterogeneous clinical manifestations might be the net result of disease burden and compensatory/reparative capacity. As a consequence, identifying what can be considered as “positive” plasticity and what, on the contrary, is a maladaptive reorganization is a very attractive goal that might help to develop therapeutic strategies able to promote the individual adaptive capacity.

This research topic provides an update on plasticity in MS. Mirroring different points of view on this topic, the collection includes a variety of different research tools, including behavioral, neurophysiological, and neuroimaging techniques, which have addressed neuroplasticity at different systems, from motor to visual and to cognitive.

Broadening our view into the cellular level, Carandini et al. review the potential role of microvesicles, i.e., spherical membrane vesicles, which are held to play a role in cell communication, in the pathogenesis of MS. Released by microglia and infiltrating macrophages, microvesicles may not only spread inflammatory signals but may also alter neuronal functions, and therefore influence synaptic plasticity.

Houdayer et al. provide a summary of the neurophysiological tools that are widely used to study cortical dysfunction in MS, with emphasis on event-related EEG oscillations, long-latency reflexes, and transcranial magnetic stimulation (TMS). In the second part, the authors present neurophysiological paradigms modulating cortical plasticity in MS, such as repetitive TMS or transcranial direct current stimulation (tDCS), which – above their great value in research – have brought some promising results as add-on treatments.

Along this line, Tecchio et al. report the effects of five sessions of tDCS targeting the bilateral whole body somatosensory area (S1wb) and the hand sensorimotor area, respectively, in 21 relapsing-remitting MS patients with fatigue. They describe a 27% reduction on the modified Fatigue Impact Scale following S1wb treatment, thereby pointing out the future therapeutic potential of non-invasive brain stimulation techniques.

Pantano et al. report findings derived from the analysis of motor network plasticity, using either active functional MRI (fMRI) tasks or resting-state investigations. The possibility of manipulating motor network plasticity by means of drugs or motor practice in order to obtain a better clinical outcome is also discussed.

How an improved understanding of the neural processes underlying functional recovery might contribute to guide rehabilitation strategies and the development of novel recovery interventions is introduced by Lipp and Tomassini.

Gallo et al. discuss the role of adaptive functional changes at the level of the visual cortex, which have mostly been assessed by photic-stimulated or resting-state fMRI following acute optic neuritis (ON). Data support an adaptive role of neuroplastic changes at the level of the occipital extrastriate cortex, which might promote visual recovery after ON. The authors speculate that models of visual plasticity might prove useful to evaluate the effect of plasticity promoting molecules.

By analyzing current discrepancies in the literature on cognitive network function (and dysfunction), Schoonheim et al. propose a model of functional reorganization, which moves from the analysis of single regions and/or networks to a more holistic network model of the entire brain, which can be explored, for instance, by using graph analysis. The need to validate this model in a longitudinal framework is also emphasized.

The notion that the use of novel approaches would provide a better understanding of the role of functional plasticity in improvement following cognitive training is also supported by the case-based fMRI series presented by Hubacher et al., who describe the occurrence of different and opposed response patterns after the same training in different subjects.

Starting from the clinico-pathologic dissociation between MS disease burden and cognitive functions, Sumowski accounts for the cognitive reserve hypothesis, which postulates that enriching life experiences protect against cognitive decline in the face of age and neurological disease. Test algorithms to identify MS patients at greatest risk for future cognitive decline may allow probing early interventions, like intellectual enrichment programs. Aside from such clinical measures, MRI-based markers will provide measurable proxies for estimating the individual reserve.

The importance of identifying adaptive versus maladaptive neuroplasticity associated with specific cognitive rehabilitation programs in MS patients with the main disease clinical phenotypes to foster the validation of the most effective cognitive rehabilitation interventions for these subjects is defended by Chiaravalloti et al.

Enzinger and Fazekas provide a critical revision of the development and current state of imaging techniques to assess MS-related morphologic damage and their contribution to understand the clinical consequences of MS (disability, and also cognitive problems and fatigue). They discuss why measurement of brain damage by structural MRI alone is not enough to comprehensively appreciate the consequences of the disease, although it is ideal for specific questions (e.g., assessment of disease activity, remyelination, or evolution of atrophy). All of this prompts toward an integrated use of structural and functional imaging techniques to assess disease progression in these patients.

Finally, Flachenecker summarizes current scientific evidence of MS rehabilitation. Given the main goal of rehabilitation therapy, i.e., facilitating adaptation and reorganization within the CNS, rehabilitation may rightly be regarded as “applied neuroplasticity.” The author points to the need of further carefully designed studies on the effectiveness of neurorehabilitation including both clinical outcomes and neuroplastic measures in order to bridge the gap between basic science and clinical experience.

This Research Topic thus offers a synopsis of recent advances of plasticity research in MS. It aims at broadening the view across systems and techniques and at stimulating further studies on this emerging and fascinating topic.

## Author Contributions

DZ, MR: editorial writing and editing.

## Conflict of Interest Statement

The authors declare that the research was conducted in the absence of any commercial or financial relationships that could be construed as a potential conflict of interest.
